# Early Surgical Intervention Followed by Antifungals in Rhino-Orbital Mucormycosis in Patients With COVID-19 Favors Clinical Outcome: A Case Series

**DOI:** 10.7759/cureus.17178

**Published:** 2021-08-14

**Authors:** Debarchan Barman Roy, Vandana Gupta, Ashutosh Biswas, Mansi Verma

**Affiliations:** 1 Medicine, All India Institute of Medical Sciences, New Delhi, IND; 2 Dentistry, All India Institute of Medical Sciences, New Delhi, IND; 3 Radiodiagnosis, All India Institute of Medical Sciences, New Delhi, IND

**Keywords:** covid-19, fungal infection, mucormycosis, cytokine, diabetes

## Abstract

Mucormycosis is an invasive fungal infection occurring in patients with or without preexisting medical illnesses. During the ongoing coronavirus disease 2019 (COVID-19) pandemic, there have been increasing reports of bacterial and fungal coinfections among some COVID-19 patients with preexisting comorbid illnesses such as diabetes and hypertension, with mucormycosis being one of them. The management of this dreaded fungal infection demands early and prompt surgical intervention to thoroughly remove the infected tissue and necrotic material to reduce the tissue burden of this invasive organism. This should be accompanied by expeditious initiation of amphotericin B along with supportive therapy. Here we present five cases of rhino-orbital mucormycosis in patients with COVID-19, all of whom presented with orbital and facial swelling (three had symptoms of impending blindness) under the backdrop of COVID-19 symptoms in the form of intermittent fever and slight dyspnea. Our treatment strategy comprised an expeditious use of early surgical intervention and amphotericin B along with the control of cytokine storm and hyperglycemia. This treatment strategy eventually resulted in an improved clinical outcome.

## Introduction

The repeated upsurge in the cases of severe acute respiratory syndrome coronavirus-2 infection, or coronavirus disease 2019 (COVID-19), is still causing havoc worldwide [1]. The presence of preexisting comorbidities such as diabetes mellitus, hypertension, hypothyroidism, chronic obstructive pulmonary disease, chronic kidney disease, chronic liver disease, and immune-compromised disorders in these patients predisposes them to develop several opportunistic infections that increase the risk of mortality [2]. Interestingly, uncontrolled diabetes has long been considered to be the most common risk factor associated with mucormycosis. Mucormycosis in association with COVID-19 may involve different body tissues, leading to rhino-orbital-cerebral, pulmonary, cutaneous, gastrointestinal, disseminated, and miscellaneous forms [3]. A review of the literature revealed cases of COVID-19 mucormycosis, with the rhino-orbital form being commonly reported [2,4].

To avoid complications of mucormycosis, early administration of antifungal medications and extensive surgical debridement based on clinical suspicion alone have been suggested by Ajith Kumar and Gupta [5]. A few studies have inferred that morbidity and mortality of rhino-orbital mucormycosis are determined by the time gap between the initiation of surgical debridement and amphotericin B coupled with the delay in controlling the underlying risk factors. The most notable complication is extension into the cerebral hemisphere which leads to poor prognosis and survival rate [6,7]. Major complications associated with this fungal illness include cavernous sinus thrombosis, carotid artery obstruction, central nervous system hemorrhage, abscess, inflammation, and permanent blindness [6].

Here, we report five cases of rhino-orbital mucormycosis associated with COVID-19, where an early clinical diagnosis followed by early surgical intervention and antifungal medications successfully reduced the mortality and morbidity and resulted in an improved clinical outcome.

## Case presentation

In view of the recent surge in COVID-19 cases in India, the general medicine ward at the All India Institute of Medical Sciences, New Delhi had to be converted into a COVID-19-care facility where we treated a few hundred patients in early 2021. A single-time, cross-sectional study was undertaken which included the patients of COVID-19 and rhino-orbital mucormycosis coinfection admitted during May 2021. Demographic details of these patients along with their previous known comorbidities and clinical presentation at admission were noted. Laboratory parameters, histopathological, microbiological, and radiological investigations, along with the treatment received during hospital stay were documented (Table 1).

**Table 1:** Clinical characteristics and investigations of the patients. IgA: Immunoglobulin A; HbA1c: glycated hemoglobin; CRP: C-reactive protein; IL-6: interleukin-6; HPE: histopathological examination; KOH: potassium hydroxide; Inj.: injection; O_2_: oxygen

Case Number	Age/Sex	Comorbidities	COVID-19 severity	Steroid use	Remdesivir use	Tocilizumab use	O_2_ requirement	Clinical presentation	Capillary blood sugar at admission (normal = 80-140 mg/dL)	HbA1_c_ (normal ≤6.5%)	Fibrinogen (normal = 220-496 mg/dL)	D-dimer (normal = 0-255 ng/mL)	CRP (normal = 1-5 mg/L)	Ferritin (normal = 15-150 ng/mL)	IL-6 (normal = 5-15 pg/mL)	HPE and fungal KOH smear showing broad aseptate hyphae	Radiological findings	Time for surgical treatment since admission	Surgical treatment	Medical treatment (3-5 mg/kg of liposomal amphotericin B)	Other treatment	Clinical outcome
1.	32/M	IgA nephropathy	Mild	Yes	No	No	No	Excruciating pain and blurring of vision in the right eye, along with nasal congestion for 7 days	208	-	845.9	370.8	114.61	4000	64.89	-	Mucosal thickening of bilateral sinuses	5 days	Endoscopic debridement under local anesthesia with conscious sedation	Inj. liposomal amphotericin B and prednisolone	Mycophenolate mofetil and tacrolimus	Improved
2.	50/F	Diabetes mellitus	Mild	No	No	No	No	Right hemicranial headache 45 days ago, followed by intermittent fever spikes and slight breathlessness for 3 days	101	7.49	321.7	384.5	20.9	1298	7.80	Yes	Mucoperiosteal thickening of bilateral sinuses	7 days	Endoscopic debridement under local anesthesia	Inj. liposomal amphotericin B, syr. posaconazole, and antibiotic cefoperazone-sulbactam	Oral antidiabetic drugs	Improved
3.	39/M	None	Mild	Yes	No	No	No	Severe pain, swelling, diminution of vision in the left eye, along with concurrent nasal crusting for the last 5 days	94	-	421.2	326.1	32.1	789.2	5.90	Yes	Mild mucosal thickening of bilateral sinuses	6 days	Endoscopic debridement under local anesthesia with conscious sedation	Inj. liposomal amphotericin B	None	Improved
4.	65/F	Diabetes mellitus, hypertension, and hypothyroidism	Mild	Yes	No	No	No	Fever, nonproductive cough, exertional dyspnea, along with left-sided gross facial swelling and nasal crusting for 7 days	197	7.9	549.5	1050	7.26	612.5	6.3	-	Left maxillary and ethmoid sinus soft tissue	8 days	Bilateral endoscopic debridement under general anesthesia	Inj. liposomal amphotericin B	Oral antidiabetic drugs, oral antihypertensive drugs, and levothyroxine	Improved
5.	62/M	Diabetes mellitus and hypertension	Mild	Yes	No	No	No	Right eye swelling with gross diminution of vision, right-sided facial swelling, nasal congestion for 5 days, along with occasional severe headache	345	9.92	587.6	387.6	148.1	553	59.87	-	Rhinosinusitis with invasion to pterygopalatine fossa and right orbital apex	7 days	Endoscopic debridement along with right eye exenteration under general anesthesia	Inj. liposomal amphotericin B	Oral antidiabetic drugs and oral antihypertensive drugs	Improved

One patient had received immunosuppressive medications due to his previous comorbid illness and subsequently developed features suggestive of rhino-orbital mucormycosis. Three out of five (60%) patients who were taking antidiabetic medications had elevated glycated hemoglobin levels at admission and demonstrated an elevated blood sugar level in the prior six-week period, which suggested improperly controlled diabetes. One patient with no known comorbidities had been diagnosed with COVID-19 one week prior and was on oral corticosteroid. Another patient had mild hypokalemia of 3.3 mmol/L. Three patients had a combination of raised blood glucose levels and elevated inflammatory markers.

**Figure 1:**
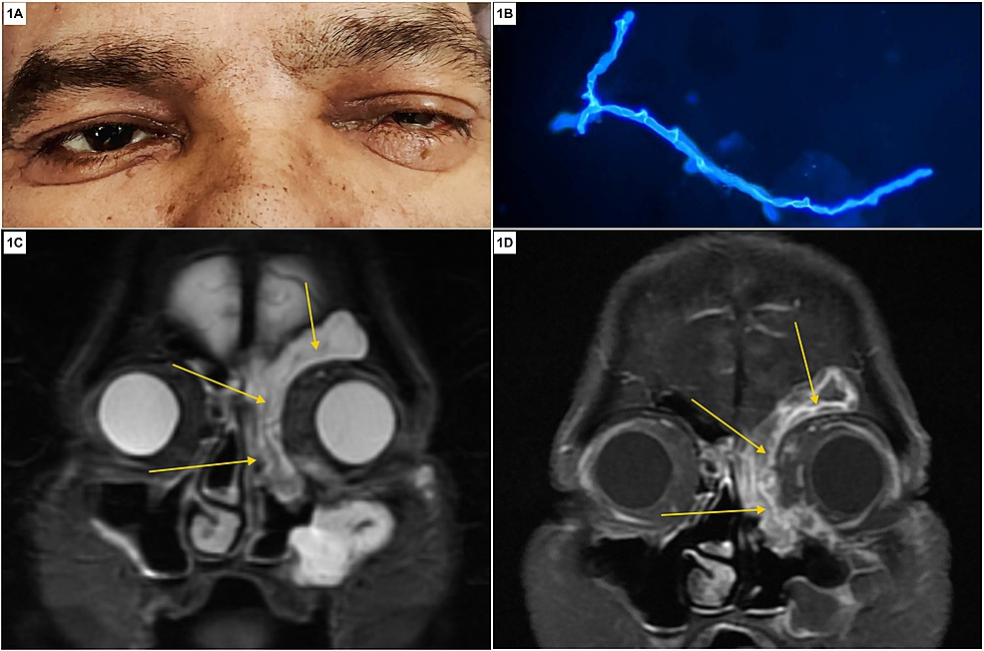
Clinical, microbiological, and radiological presentation of COVID-19 rhino-orbital mucormycosis. (A) Patient with left eye swelling associated with pain and diminution of vision in the same eye for five days, suggestive of orbital involvement due to mucormycosis. (B) Calcofluor white–KOH mount visualized under fluorescence microscopy (400× magnification) showing the presence of broad ribbon-like aseptate hyphae with branching at 90 degrees in the nasal debridement tissue sample, suggestive of invasive mucormycosis. (C) Coronal T2-weighted image depicting mucosal thickening and hyperintense soft tissue (shown with yellow arrows) in the left frontal, ethmoid, maxillary sinus, and medial part of the left orbit. (D) Coronal postcontrast T1 image depicting heterogeneous enhancement of the soft tissue (shown with yellow arrows) in various paranasal sinuses. COVID-19: coronavirus disease 2019

Mucormycosis was diagnosed concurrently in these patients by examining their clinical presentation in conjunction with radiological, histopathological, and microbiological findings. The median age of our patients was 50 years (ranging from 32 to 65 years). Excruciating orbital pain, visual blurring, dimness of vision, facial pain, facial swelling, nasal congestion, and fever were the common presenting complaints of all five patients. Notably, three (60%) patients had diminution of vision whereas one presented with complete loss of vision (Figure 1A). In addition, two patients had foul-smelling, nasal crusting while one complained of occasional severe headaches. Nasal scraping was collected from all five patients at the time of admission, of which only two samples revealed broad aseptate fungal hyphae with branching (Figure 1B). On magnetic resonance imaging, all patients had mucosal thickening and soft tissue involvement of multiple paranasal sinuses (Figure 1C, 1D).

All patients were treated similarly with early endoscopic sinus surgery for debridement of the infected and necrotic tissue, with the mean duration to debridement being six days since admission. Additionally, intravenous liposomal amphotericin B and other supportive treatments for COVID-19 were started immediately. Our strategy was to decrease the burden of the fungus in the affected tissues to avoid further spread and associated complications. One patient underwent orbital exenteration of the affected eye under general anesthesia. All five patients tolerated the therapy well; however, the patient with preexisting hypokalemia required close monitoring of serum potassium and frequent intravenous supplementation. Three patients had elevated blood glucose levels and were treated with subcutaneous insulin injections.

Eventually, all patients improved with treatment and were shifted out to the non-COVID-19 ward after they were tested negative for COVID-19 to complete the antifungal course. Once the induction phase of liposomal amphotericin B was over, syrup posaconazole was started and continued.

## Discussion

The COVID-19 pandemic is still raging worldwide claiming lives and shattering preconceived notions. As humanity strives to survive compiling data from days and nights of hard work and research to discover a potential cure against this unseen enemy, no current specific treatment exists for this disease. Glucocorticoids, be it oral, intravenous, or inhaled, are being considered indispensable in this war against COVID-19 and have proven to be a useful therapy in reducing prolonged hospital stay as well as mortality and morbidity in severely ill COVID-19 patients [8]. Although the rampant use of glucocorticoids increases the risk of secondary infections, both bacterial and fungal, which is well established in the literature [9], the potential association between COVID-19 and increased fungal infections needs further scientific evidence. One such dreaded infection is mucormycosis with at least six different clinical presentations: rhino-orbital-cerebral, pulmonary, cutaneous, gastrointestinal, disseminated, and miscellaneous. Among these presentations, rhino-orbital-cerebral is considered to be the most common manifestation, followed by pulmonary and cutaneous presentations [7]. The fungus usually gains entry into the host through the respiratory tract and exhibits a remarkable affinity for the arteries, growing along their internal elastic lamina and causing thrombosis and infarction [10]. The progression of the disease from the nose and sinuses is either direct or through vascular invasion. Intracranial involvement may also occur by invasion through the superior orbital fissure, ophthalmic vessels, cribriform plate, and carotid artery or via the perineural route [11,12]. The diagnosis of this disease entity should be based on the clinical features with the support of microbiological and radiological findings [13]. Waiting for cultures is impractical and may lead to an inadvertent delay in the initiation of treatment. If a clear clinical picture of mucormycosis exists, positive KOH mounts may be sufficient for initiating treatment [14]. In our series, three out of five patients did not yield any fungal elements on the KOH mount prepared using the nasal scrapings. However, considering the clinical presentation and keeping in mind the possibility of uncontrolled blood glucose levels with preexisting immunocompromised status, a clinical diagnosis of mucormycosis was considered.

Furthermore, a multiplanar MRI which shows anatomic involvement may be used to plan surgical interventions [15]. Early establishment of the diagnosis and prompt surgical intervention should be targeted to control any further extension of the disease. The imaging studies undertaken in our patients showed similar results and were utilized to support our opinion regarding the clinical diagnosis and thus helped in our clinical decision-making.

In our series, three out of five patients had uncontrolled capillary blood glucose levels and elevated glycated hemoglobin levels, while two out of these three patients had a combination of both elevated blood glucose levels and elevated inflammatory markers. As pointed out by John et al., such patients having a confluence of both diabetic and COVID-19 storm are at an increased risk for mucormycosis; hence, they suggested keeping a high clinical suspicion in similar patients [16]. Moreover, control of hyperglycemia should assume greater importance than preemptive antifungal therapy in such patients. The primary guideline for managing this disease is to correct the underlying cause, but this may not be immediately achieved in patients who are dependent on high-dose corticosteroid therapy like in cases with active COVID-19 infection. The existing recommendations for mucormycosis treatment suggest high-dose lipid formulations of amphotericin B or isavuconazole as the first-line therapy and posaconazole (oral suspension or newer formulations such as intravenous injections or extended-release tablets) as salvage therapy [17]. However, the importance of early and prompt surgical debridement has been stressed by Sharma et al. in a study where all 23 patients who underwent the surgical procedure survived on follow-up [18].

Hyperbaric oxygen therapy and local treatment involving the initiation of amphotericin B may be used as adjunctive modalities of treatment. Amphotericin B is a fungistatic agent rather than a fungicidal agent and may lead to a prolonged treatment duration [19]. The use of oral posaconazole may be considered as an additional add-on/alternative therapy for rhino-orbital mucormycosis in COVID-19 [20].

## Conclusions

Early diagnosis of COVID-19 mucormycosis through sharp clinical acuity and initiation of early surgical intervention seems to be the key to control this disease burden and prevent its numerous debilitating complications. Prompt initiation of antifungals along with control of the cytokine storm and diabetes should be the next concern. In such patients, we suggest early surgical debridement within five to eight days of the establishment of the diagnosis to reduce the burden of the fungus within the infected body tissue and to reduce further morbidity and mortality. We hope during further encounters with COVID-19 mucormycosis coinfections, clinicians would be better armed to initiate early therapy and achieve a favorable clinical outcome among their patients.
